# Supplementary material for: An analysis of pacing profiles in sprint kayak racing using functional principal components and hidden Markov models
Source: PLoS One. 2025 Jul 2;20(7):e0326375. doi: 10.1371/journal.pone.0326375 (PMC12221036; doi:10.1371/journal.pone.0326375)
Supplement: S1 File — Appendix 1. AIC Values calculated for different number of states in HMM. Appendix 2. Standard Deviation for each state and principal component in the HMM. (DOCX) [file pone.0326375.s001.docx]

# Appendix

### Appendix 1

AIC Values calculated for different number of states in HMM

| **No. of States** | **AIC** |
| --- | --- |
| **2** | -934 |
| **3** | -1049 |
| **4** | -1121 |
| **5** | -1124 |
| **6** | -1135 |
| **7** | -1144 |

### Appendix 2

Standard Deviation for each state and principal component in the HMM

| **Women’s K1 500m** | | | | |  | **Men’s K1 1000m** | | | |
| --- | --- | --- | --- | --- | --- | --- | --- | --- | --- |
|  | **PC1** | **PC2** | **PC3** | **PC4** |  | **PC1** | **PC2** | **PC3** | **PC4** |
| **State 1** | 0.173 | 0.230 | -0.003 | -0.028 |  | -0.871 | 0.182 | -0.126 | -0.137 |
| **State 2** | -0.418 | 0.552 | -0.136 | 0.168 |  | 0.979 | 0.287 | 0.030 | -0.068 |
| **State 3** | -0.373 | -0.105 | 0.070 | 0.050 |  | -0.107 | 0.654 | 0.080 | 0.185 |
| **State 4** | 0.063 | -0.045 | -0.045 | -0.039 |  | 0.118 | 0.007 | -0.023 | 0.030 |

### Appendix 3

Principal Component distributions for both the Men’s and Women’s dataset. There is also an example fitdistrplus plot for Men’s PC4 validating that the distribution is Geometric.

### Appendix 4

Histogram of Sojourn times and fitdistrplus plot for both Men’s and Women’s dataset

### Appendix 5

Example histogram of residuals for state 1 in the Men’s HMM and a example QQ plot analysis for PC4.
