# Supplementary figures and images for: An analysis of pacing profiles in sprint kayak racing using functional principal components and hidden Markov models
Source: PLoS One. 2025 Jul 2;20(7):e0326375. doi: 10.1371/journal.pone.0326375 (PMC12221036; doi:10.1371/journal.pone.0326375)

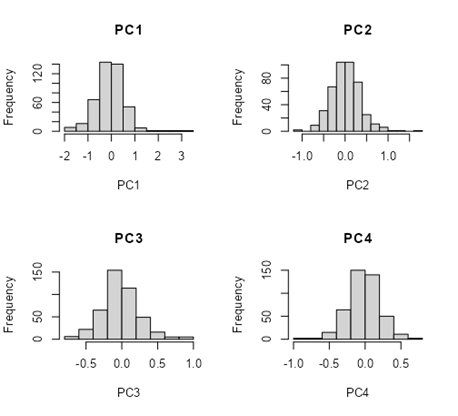

Supplement: S1 Fig — Appendix 3. Principal Component distributions for both the Men’s and Women’s dataset. There is also an example fitdistrplus plot for Men’s PC4 validating that the distribution is Geometric. Appendix 4. Histogram of Sojourn times and fitdistrplus plot for both Men’s and Women’s dataset. Appendix 5. Example histogram of residuals for state 1 in the Men’s HMM and a example QQ plot analysis for PC4. (ZIP) [file pone.0326375.s002.zip › Appendix 3.1.tif]

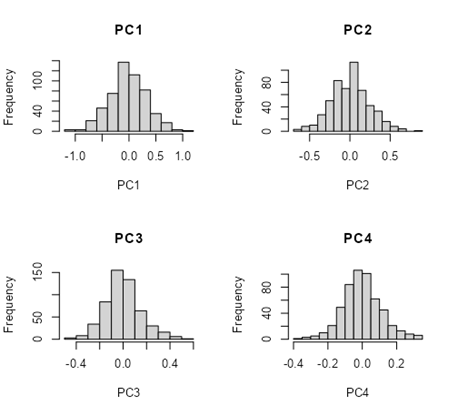

Supplement: S1 Fig — Appendix 3. Principal Component distributions for both the Men’s and Women’s dataset. There is also an example fitdistrplus plot for Men’s PC4 validating that the distribution is Geometric. Appendix 4. Histogram of Sojourn times and fitdistrplus plot for both Men’s and Women’s dataset. Appendix 5. Example histogram of residuals for state 1 in the Men’s HMM and a example QQ plot analysis for PC4. (ZIP) [file pone.0326375.s002.zip › Appendix 3.2.tif]

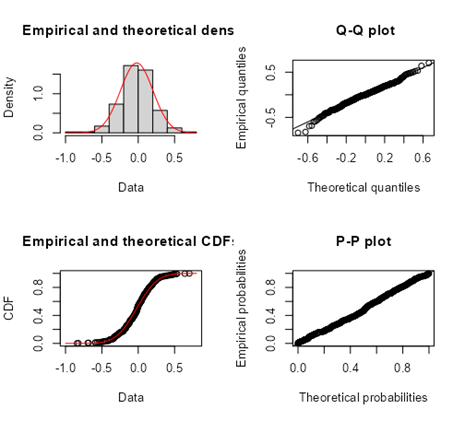

Supplement: S1 Fig — Appendix 3. Principal Component distributions for both the Men’s and Women’s dataset. There is also an example fitdistrplus plot for Men’s PC4 validating that the distribution is Geometric. Appendix 4. Histogram of Sojourn times and fitdistrplus plot for both Men’s and Women’s dataset. Appendix 5. Example histogram of residuals for state 1 in the Men’s HMM and a example QQ plot analysis for PC4. (ZIP) [file pone.0326375.s002.zip › Appendix 3.3.tif]

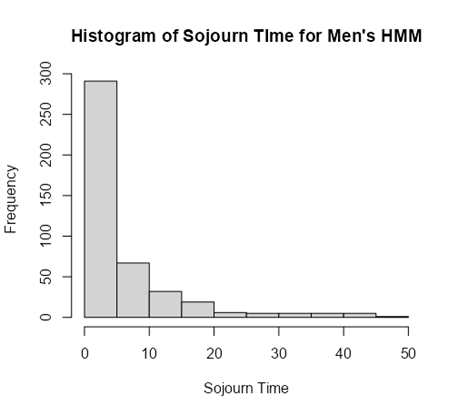

Supplement: S1 Fig — Appendix 3. Principal Component distributions for both the Men’s and Women’s dataset. There is also an example fitdistrplus plot for Men’s PC4 validating that the distribution is Geometric. Appendix 4. Histogram of Sojourn times and fitdistrplus plot for both Men’s and Women’s dataset. Appendix 5. Example histogram of residuals for state 1 in the Men’s HMM and a example QQ plot analysis for PC4. (ZIP) [file pone.0326375.s002.zip › Appendix 4.1.tif]

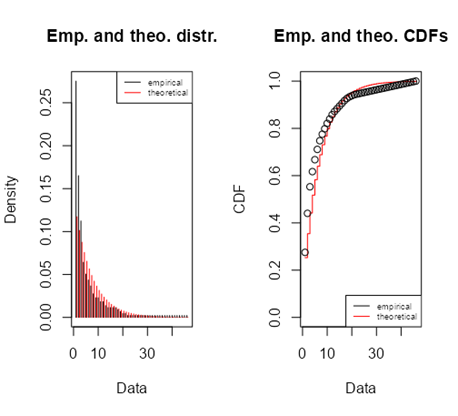

Supplement: S1 Fig — Appendix 3. Principal Component distributions for both the Men’s and Women’s dataset. There is also an example fitdistrplus plot for Men’s PC4 validating that the distribution is Geometric. Appendix 4. Histogram of Sojourn times and fitdistrplus plot for both Men’s and Women’s dataset. Appendix 5. Example histogram of residuals for state 1 in the Men’s HMM and a example QQ plot analysis for PC4. (ZIP) [file pone.0326375.s002.zip › Appendix 4.2.tif]

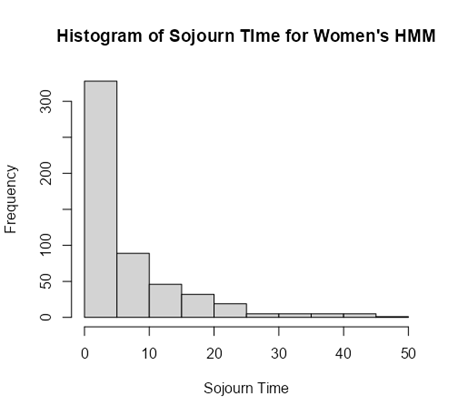

Supplement: S1 Fig — Appendix 3. Principal Component distributions for both the Men’s and Women’s dataset. There is also an example fitdistrplus plot for Men’s PC4 validating that the distribution is Geometric. Appendix 4. Histogram of Sojourn times and fitdistrplus plot for both Men’s and Women’s dataset. Appendix 5. Example histogram of residuals for state 1 in the Men’s HMM and a example QQ plot analysis for PC4. (ZIP) [file pone.0326375.s002.zip › Appendix 4.3.tif]

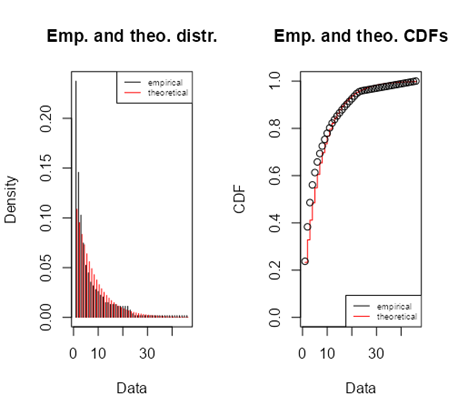

Supplement: S1 Fig — Appendix 3. Principal Component distributions for both the Men’s and Women’s dataset. There is also an example fitdistrplus plot for Men’s PC4 validating that the distribution is Geometric. Appendix 4. Histogram of Sojourn times and fitdistrplus plot for both Men’s and Women’s dataset. Appendix 5. Example histogram of residuals for state 1 in the Men’s HMM and a example QQ plot analysis for PC4. (ZIP) [file pone.0326375.s002.zip › Appendix 4.4.tif]

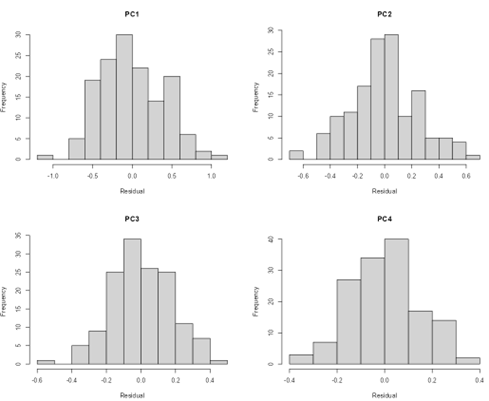

Supplement: S1 Fig — Appendix 3. Principal Component distributions for both the Men’s and Women’s dataset. There is also an example fitdistrplus plot for Men’s PC4 validating that the distribution is Geometric. Appendix 4. Histogram of Sojourn times and fitdistrplus plot for both Men’s and Women’s dataset. Appendix 5. Example histogram of residuals for state 1 in the Men’s HMM and a example QQ plot analysis for PC4. (ZIP) [file pone.0326375.s002.zip › Appendix 5.1.tif]

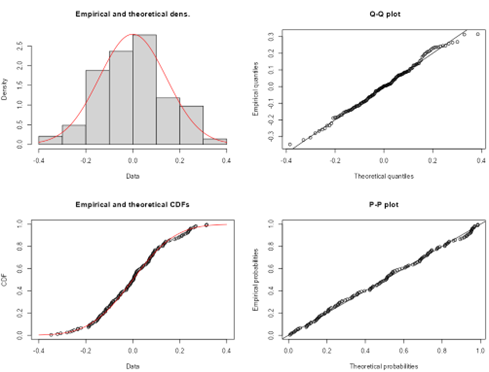

Supplement: S1 Fig — Appendix 3. Principal Component distributions for both the Men’s and Women’s dataset. There is also an example fitdistrplus plot for Men’s PC4 validating that the distribution is Geometric. Appendix 4. Histogram of Sojourn times and fitdistrplus plot for both Men’s and Women’s dataset. Appendix 5. Example histogram of residuals for state 1 in the Men’s HMM and a example QQ plot analysis for PC4. (ZIP) [file pone.0326375.s002.zip › Appendix 5.2.tif]
