# Supplementary material for: An analysis of pacing profiles in sprint kayak racing using functional principal components and hidden Markov models
Source: PLoS One. 2025 Jul 2;20(7):e0326375. doi: 10.1371/journal.pone.0326375 (PMC12221036; doi:10.1371/journal.pone.0326375)
Supplement: Attachment — Submitted filename: Response to Reviewers.docx [file pone.0326375.s005.docx]

PONE-D-24-05983R1

An analysis of pacing profiles in sprint kayak racing using functional principal components and Hidden Markov Models

PLOS ONE

Dear Dr. Estreich,

Thank you for submitting your manuscript to PLOS ONE. After careful consideration, we feel that it has merit but does not fully meet PLOS ONE’s publication criteria as it currently stands. Therefore, we invite you to submit a revised version of the manuscript that addresses the points raised during the review process.

Please submit your revised manuscript by Jan 10 2025 11:59PM. If you will need more time than this to complete your revisions, please reply to this message or contact the journal office at [plosone@plos.org](mailto:plosone@plos.org). When you're ready to submit your revision, log on to [https://www.editorialmanager.com/pone/](https://aus01.safelinks.protection.outlook.com/?url=https%3A%2F%2Furldefense.com%2Fv3%2F__https%3A%2F%2Fwww.editorialmanager.com%2Fpone%2F__%3B!!NVzLfOphnbDXSw!DGHKXYlvlGxgBQTA5QU0J-9SVt1uSd_ieHrcwP_1TJHr1-NJTrBcEsa6tfh5ps_-dXMNj7izam484gZtE1xSQz3kYQ%24&data=05%7C02%7Charry.estreich%40hdr.qut.edu.au%7C640bcf46032849c9946308dd0e3b7750%7Cdc0b52a368c544f7881d9383d8850b96%7C0%7C0%7C638682370795018298%7CUnknown%7CTWFpbGZsb3d8eyJFbXB0eU1hcGkiOnRydWUsIlYiOiIwLjAuMDAwMCIsIlAiOiJXaW4zMiIsIkFOIjoiTWFpbCIsIldUIjoyfQ%3D%3D%7C0%7C%7C%7C&sdata=Zq9hnuOMFwG3wk1fUU5CHyK%2FRHi6LRQ1B1aS3qQUmSA%3D&reserved=0) and select the 'Submissions Needing Revision' folder to locate your manuscript file.

Please include the following items when submitting your revised manuscript:

- A rebuttal letter that responds to each point raised by the academic editor and reviewer(s). You should upload this letter as a separate file labeled 'Response to Reviewers'.
- A marked-up copy of your manuscript that highlights changes made to the original version. You should upload this as a separate file labeled 'Revised Manuscript with Track Changes'.
- An unmarked version of your revised paper without tracked changes. You should upload this as a separate file labeled 'Manuscript'.

If you would like to make changes to your financial disclosure, please include your updated statement in your cover letter. Guidelines for resubmitting your figure files are available below the reviewer comments at the end of this letter.

If applicable, we recommend that you deposit your laboratory protocols in protocols.io to enhance the reproducibility of your results. Protocols.io assigns your protocol its own identifier (DOI) so that it can be cited independently in the future. For instructions see: [https://journals.plos.org/plosone/s/submission-guidelines#loc-laboratory-protocols](https://aus01.safelinks.protection.outlook.com/?url=https%3A%2F%2Furldefense.com%2Fv3%2F__https%3A%2F%2Ftrack.editorialmanager.com%2FCL0%2Fhttps%3A*2F*2Fjournals.plos.org*2Fplosone*2Fs*2Fsubmission-guidelines*23loc-laboratory-protocols%2F1%2F010f019369685105-ecbf3210-575a-4a50-aafc-c3cc0db92578-000000%2F9teAF2Tp_pjVS6VszNQnHcIoWyOlznd5d5FnQK1g8n8%3D186__%3BJSUlJSUl!!NVzLfOphnbDXSw!DGHKXYlvlGxgBQTA5QU0J-9SVt1uSd_ieHrcwP_1TJHr1-NJTrBcEsa6tfh5ps_-dXMNj7izam484gZtE1w2Q9sRAA%24&data=05%7C02%7Charry.estreich%40hdr.qut.edu.au%7C640bcf46032849c9946308dd0e3b7750%7Cdc0b52a368c544f7881d9383d8850b96%7C0%7C0%7C638682370795038888%7CUnknown%7CTWFpbGZsb3d8eyJFbXB0eU1hcGkiOnRydWUsIlYiOiIwLjAuMDAwMCIsIlAiOiJXaW4zMiIsIkFOIjoiTWFpbCIsIldUIjoyfQ%3D%3D%7C0%7C%7C%7C&sdata=lbq%2FOCb7BfuCqlYE2T8HvGeNNAkZdS%2BuCo%2BnqLbtO0M%3D&reserved=0). Additionally, PLOS ONE offers an option for publishing peer-reviewed Lab Protocol articles, which describe protocols hosted on protocols.io. Read more information on sharing protocols at [https://plos.org/protocols?utm_medium=editorial-email&utm_source=authorletters&utm_campaign=protocols](https://aus01.safelinks.protection.outlook.com/?url=https%3A%2F%2Furldefense.com%2Fv3%2F__https%3A%2F%2Ftrack.editorialmanager.com%2FCL0%2Fhttps%3A*2F*2Fplos.org*2Fprotocols*3Futm_medium%3Deditorial-email*26utm_source%3Dauthorletters*26utm_campaign%3Dprotocols%2F1%2F010f019369685105-ecbf3210-575a-4a50-aafc-c3cc0db92578-000000%2FcEyX7lG-XYGBSB9ULCcpNTJ3Uhm7LfXoIaVRde-8oDs%3D186__%3BJSUlJSUl!!NVzLfOphnbDXSw!DGHKXYlvlGxgBQTA5QU0J-9SVt1uSd_ieHrcwP_1TJHr1-NJTrBcEsa6tfh5ps_-dXMNj7izam484gZtE1xQWQfAkg%24&data=05%7C02%7Charry.estreich%40hdr.qut.edu.au%7C640bcf46032849c9946308dd0e3b7750%7Cdc0b52a368c544f7881d9383d8850b96%7C0%7C0%7C638682370795052479%7CUnknown%7CTWFpbGZsb3d8eyJFbXB0eU1hcGkiOnRydWUsIlYiOiIwLjAuMDAwMCIsIlAiOiJXaW4zMiIsIkFOIjoiTWFpbCIsIldUIjoyfQ%3D%3D%7C0%7C%7C%7C&sdata=uA5WG5ZD%2BidjaUpGMW2uEBwwybsgIl59VhDL0%2FgqYcs%3D&reserved=0).

We look forward to receiving your revised manuscript.

Kind regards,

Matteo Vandoni
Academic Editor
PLOS ONE

Replies to the Editor:

Thank you for considering our manuscript for publication in PLOS ONE.

We would like to extend our gratitude to the reviewers for their valuable feedback.

In response to the reviewers' comments and suggestions, we have made significant revisions to the manuscript. Each point raised has been carefully addressed, and our responses have been provided in blue font for clarity. Additionally, as suggested, we have highlighted the main modifications made in red throughout the manuscript to facilitate the review process.

We trust that the revisions adequately address the concerns raised by the reviewers and editors.

Thanks

Harry Estreich

School of Mathematical Sciences, Queensland University of Technology, Brisbane, QLD, Australia
Centre for Data Science, Queensland University of Technology, Brisbane, QLD, Australia

Nicola Bullock

Paddle Australia, Gold Coast, QLD, Australia
Australian Institute of Sport, Gold Coast, QLD, Australia
Bond Institute of Health and Sport, Bond University, Gold Coast, QLD, Australia

Mark Osborne

Paddle Australia, Gold Coast, QLD, Australia

Edgar Santos-Fernandez

School of Mathematical Sciences, Queensland University of Technology, Brisbane, QLD, Australia
Centre for Data Science, Queensland University of Technology, Brisbane, QLD, Australia

Paul Wu

School of Mathematical Sciences, Queensland University of Technology, Brisbane, QLD, Australia
Centre for Data Science, Queensland University of Technology, Brisbane, QLD, Australia

Reviewer #1 Feedback:

Thank you for carefully reviewing our manuscript and for the useful suggestions.

**1. As I mention before, the two-step analysis leads to misleading inference. Acknowledge this as a limitation of the study is not sufficient, as results may be unreliable if a joint approach is neglected. Moreover, it is rather unclear to me what the authors mean with "Our results have been shown to be robust in preliminary sensitivity analyses", more details on this are required.**

Thank you for further clarification, we agree that the decoupled two-stage approach – functional PCA followed by HMM – does not fully account for uncertainty propagation between stages. This is a well-recognised limitation in the literature and we now acknowledge this more explicitly in the manuscript.

However, as this is less explored with fPCA and HMMs, it is an avenue for future investigation and outside the scope of this study due to the significant increase in complexity and issues with interpretability. (Page 14)

However, computational constraints and practical considerations limit the feasibility of implementing the fully integrated approach suggested by the reviewer. To assess the extent to which this may impact our inference, we conducted RMSE validation on each principal component and reported it on Page 14.

The RMSE was less than 0.5 for each principal component which was less than the standard deviation of that component. (Page 14)

**2. I appreciate that more details on the HMM specification have been added to the main text. Are the Gaussian conditional densities with state-specific variances? If so, do you encounter any degeneracy issues? Moreover, please provide evidence that the Gaussian distribution is suited for the data at hand; the idea of removing outliers is questionable.**

Thank you for the additional clarification around your comments. As the Gaussian conditional densities have state-specific variances, we have added a table to the Appendix and added a reference to it in the text.

Each Gaussian distribution also has a state-specific variance (see Appendix 2). (Page 8)

We have checked for degeneracy issues , including with regards to collapsed states and state redundancy, and reported the outcome Page 14 with results in Table 4

Degeneracy issues were also checked, and there we no collapsed states as the transition matrix (Table 4) allows for transitions out of every state, and emission matrices (Tables 1 through 3) show that no two states are the same (i.e. not redundant). (Page 14)

I have also added evidence that the Gaussian distribution is suitable by adding principal component distributions and a fitdistrplus example.

This was verified by checking the distributions of each principal component using fitdistrplus package (see Appendix 3). (Page 14)

Lastly, I have clarified how we removed the outliers.

Outlier removal was conducted based on input from domain experts, who leveraged their deep understanding of the data-generating process to identify observations that were inconsistent with the expected patterns or operational constraints of the system. This expert-driven approach ensured that the outliers identified were truly anomalous and not representative of the underlying phenomena being studied. (Page 4)

**3. Residual analysis, qq-plot graphs, etc should be shown to ensure that the model is suitable for the data at hand. The AIC, and other model selection criteria, are useful to select the number of clusters (and to compare different model specifications) but not to guarantee that the model is adequate for the data at hand.**

Thank you for this clarify comment. I have added additional residual analysis and qq-plots. I have added an example of each plot below. Based off these, there is no evidence to reject the assumption that the PCs follow a normal distribution.

Residual analysis also confirmed that the model is suitable for the data. (Page 8)

**4. At last, one further point must be discussed and investigate, via models comparison. The HMM implicitly assume that the sojourn distribution is geometric. Please, check that this assumption is met and relax it if the case by assuming e.g. shifted negative binomial, logarithmic, etc sojourns. Please, provide evidence that the geometric sojourn is chosen according to any model selection criteria like the AIC.**

Thank you for your comment, I have explicitly stated the test used (goodness-of-fit test on geometric distribution) and added the tests to the appendix. This was used as justification to assume the sojourn distribution is geometric.

Additionally, we calculated the empirical distribution of the sojourn times for each hidden state and compared them to the geometric distribution using goodness-of-fit tests (this was done using the R package fitdistrplus) (see Appendix 4) (Page 8)
